# Assessment of the Possible Correlation between the Presence of Helicobacter Pylori Infection and Hairy Tongue Lesion in a Group of Patients in Syria: A Cross-Sectional and Pilot Study

**DOI:** 10.3390/ijerph20021324

**Published:** 2023-01-11

**Authors:** Dania Sawan, Ammar M. Mashlah, Mohammad Younis Hajeer, Abeer A. Aljoujou

**Affiliations:** 1 Department of Oral Medicine, College of Dentistry, Damascus University, Damascus MY1 HAJ72, Syria; 2 Department of Orthodontics, College of Dentistry, Damascus University, Damascus MY1 HAJ72, Syria

**Keywords:** helicobacter pylori, hairy tongue, serological tests, *H-pylori* IgG antibodies

## Abstract

Background: This study aimed to evaluate the correlation between the presence of hairy tongue and *H. pylori* infection in patients referring to their blood test based on the serum levels of anti-*H pylori* IgG antibodies. Methods: This cross-sectional study was conducted in the Department of Oral Medicine, University of Damascus Dental School, between February 2021 and January 2022. The sample size of 40 patients (23 males, 17 females), whose ages ranged from 20–79 years with a mean age of 41.5 ± 12 years, was calculated using the G*power 3.1.3, with a statistical power of 80% and a significance level of 0.05. The hairy tongue index was assessed by a visual method based on observing the dorsum tongue appearance. Then, a blood test was performed to detect the presence of *H. pylori* by Immulite 2000 XPi. Statistical analysis was performed using SPSS software 22.0, Chi-square. Results: The prevalence of hairy tongue was higher among males (75%) as compared to females (25%) and was found to be statistically significant (*p* = 0.026). The hairy tongue lesions were found to be least in the 20–39 age group and most prevalent in the 40–59 age group, without statistically significant correlation. *H. pylori* infection was detected positive in 70% and negative in 30% of hairy tongue patients, compared to the control group, where the rates were 15% and 85%, respectively, with a statistically significant correlation between infection with *H. pylori* and hairy tongue (*p* = 0.001). Conclusion: Our results strongly suggest that the hairy tongue might be considered an indicator of *H. pylori* infection.

## 1. Introduction

The tongue is a vital organ in the oral cavity; it acts as an index for underlying systemic diseases [1]. Systemic diseases may first present with alterations in the tongue [2]. Traditionally, the tongue performs an essential function in preserving concord inside the oral environment [3]. The tongue is essentially a gateway to the digestive system [4].

The tongue manifestations represent the characteristics of the pathogenic factors and the state of the internal organs, especially the spleen and stomach [5]. Furthermore, the color of the middle of the tongue body indicates acute changes in the gastric mucosa and is a useful noninvasive screening tool for erosive gastritis [6]. A hairy tongue is one of these lesions caused by a lack of adequate desquamation of keratin over the filiform papillae [7]. The discoloration is caused by porphyrin-producing chromogenic bacteria or yeast [8]. Defective desquamation prevents normal debridement ensuing in an immoderate increase and thickening of the filiform papillae that collect debris, bacteria, fungi, or other foreign materials, all of which contribute to the discoloration [9]. The shadeation stages from cream to brown to black, relying on extrinsic factors, inclusive of diet and smoking, and intrinsic factors, such as chromogenic macro-organisms and fungi [10].

The oral microbiome helps the host against the invasion of opportunistic microorganisms. The oral microbiome also impacts the microbial communities that colonize the gastrointestinal tract, and its imbalances not only contribute to oral diseases but also the risk of gastrointestinal disorders, adverse pregnancy outcomes, cardiovascular disease, diabetes, rheumatoid arthritis, and systemic nervous diseases [11].

Xing Se Joan Mo stated that “the tongue coating is shaped via way of means of the stomach”. In clinical studies, researchers have mentioned that the tongue frame modifies slowly with the improvement of chronic gastritis, but the tongue coating changes rapidly and obviously [7]. Observing the researched tongue-coating metabolic markers and the composition of microorganisms in persistent gastritis sufferers helps to determine whether there is any relationship between tongue diagnosis (noninvasive detection) and metabolic processes or microorganisms [5]. Some of these microorganisms are microaerophilic, Gram-negative bacterium whose primary ecological niche is the human stomach, such as Helicobacter pylori [12]. Helicobacter pylori *(H. pylori*) is a spiral-shaped bacterium that grows in the digestive tract and can be found in more than half of the world’s population. The clinical features of Heliobacter pylori vary from symptomless inflammation to gastrointestinal malignancy [13]. The pathogenic properties of *H. pylori* include its ability to survive in gastric juice, reduce gastric acidity, and colonize the gastric mucosa [12]. The detection and isolation of *H. pylori* from the gastric mucosa have been carried out successfully. However, reviews of extra-gastric isolation, primarily in the oral cavity, are nevertheless controversial [14]. Additionally, the influence of local and gastric infections on the development of oral pathology is unclear [15].

A wide range of laboratory investigations are available to identify *H. pylori*. These are both noninvasive and invasive tests. Noninvasive tests include serology, a urea breath test, and a stool antigen test. Invasive tests include histology, rapid urease test and culture [16]. Blood antibody tests detect the presence of antibodies of *H. pylori* in the upper part of the small intestine (duodenum) and stomach [17]. However, immunoglobulin (IgG) antibodies can persist in the blood for years after the bacteria have been completely eradicated as serological tests simply detect an immune response [18]. In epidemiological studies, serum tests could offer a high sensitivity of 88–94% and a specificity of 74–88% [17]. Serum assaying of anti-*H pylori* IgG antibodies could be used to determine the prevalence of acute and chronic infections [19]. In general, the serum levels of anti-*H pylori* IgG antibodies increase in the presence of infection and could be used as a marker [14].

A study by Hou et al. showed that tongue manifestations could be a feasible predictor of *H. pylori* infection in patients with gastrointestinal symptoms. Hou et al. reported a significant difference in the prevalence of *H. pylori* infection between patients with thick and thin coatings in yellow tongue compared to white tongue patients [20]. In other studies, there was a higher risk of *H. pylori* infection with coated tongue, such as the study of Mohammadi et al., which determined the prevalence of *H. pylori* infections and its relationship with coated tongue in the patients referred for a urease breath test. It was revealed that 41.1% of the patients with coated tongue had *H. pylori* infection, whereas this value was 22.7% in the participants with non-coated tongue had *H. pylori* infection. The findings showed a significant relationship between coated tongue and *H. pylori* infection (*p* = 0.025) [21]. Moreover, Zaric et al. showed an association between *H. pylori* infection and coated tongue; they observed coated tongue in 18 patients (27.2%) out of 66 who had gastric *H. pylori* infection and in only 2 patients (6.25%) out of 32 without *H. pylori* infection (*p* = 0.0164). Their findings suggested that the eradication of gastric *H. pylori* significantly alleviated coated tongue, which may be considered as an extra gastric manifestation of this common chronic *H. Pylori* infection [12].

Reviewing the literature has revealed no research on the relationship between hairy tongue and *H. pylori* [12,21]. All the published articles evaluating coated tongue were only confined to oral manifestations of some lesions and studied its relationship with H. Pylori infection using invasive tests. The test has been used in Mohammedi’s study, which was a urease breath test [21], and Zaric’s study using an invasive biopsy gastric test [12], whereas Hou et al. did not mention the name of the test they used. It is recommended to conduct more clinical studies with a noninvasive serological test to evaluate the presence of *H. pylori* infection in its acute and chronic states, regardless of the treatment used or unused.

This study aimed to evaluate the correlation between the presence of hairy tongue and *H. pylori* colonization in patients referring to their blood test based on the serum levels of anti-*H pylori* IgG antibodies.

## 2. Materials and Methods

### 2.1. Study Design and Settings

This study was designed as a cross-sectional study. It was conducted in the Department of Oral medicine, University of Damascus Dental School between February 2021 and January 2022. Ethical approval was obtained from the Local Research Ethics Committee at the Faculty of Dentistry of the University of Damascus (UDDS-236-12012016/SRC-1552). 

### 2.2. Sample Size Calculation

The sample size was calculated using the G*power 3.1.3 program (Heinrich-Heine-University, Düsseldorf, Germany) with a statistical power of 80%, and a significance level of 0.05. It was assumed that the detection rates of *H. pylori* in the symptomatic and asymptomatic oral lesions groups were 87.3% and 49.5%, respectively, in a previous study [22]. A difference in the proportion of 40% was used to calculate the required sample size. Employing the chi-square test, the required sample size was 20 patients in each group. The total number of patients required for both groups was 40 patients.

According to previous studies, the prevalence of hairy tongue varies geographically, typically ranging from 5.3% to 11.3% in Arabic countries or among the Arabic population [23,24,25]. Therefore, the examination of more than 377 outpatients referred to the Oral Medicine Department was suggested to obtain the required number of candidate patients.

### 2.3. Sampling Frame and Patients’ Recruitment

After examining 400 patients attending the Department of Oral medicine at the University of Damascus, it was found that 32 patients (20–79 years) met the inclusion criteria. The research project was clarified to the patients. Among the 27 patients who agreed to join the study, 20 (15 males and five females) were randomly selected. By all examined patients, it was found that 210 patients (20–79 years) met the conditions for inclusion into the control group (non-hairy tongue patients). Among the 190 patients who agreed to join the study, 20 (8 males and 12 females) were randomly selected from the sampling frame and were included in the study. The project took place between February 2021 and January 2022.

Information sheets were given to all patients, and informed consent forms were collected upon approval.

The inclusion criteria were the following: (1) patients with hairy tongue lesions assessed clinically; (2) no general problems; (3) good oral health; (4) age between 20 and 79 years. The exclusion criteria involved the following: (1) the presence of other oral diseases; (2) the presence of severe systemic diseases; (3) the use of immunosuppressive drugs; (4) the patient refused the process of study; (5) children and adolescents; (6) pregnant and lactating women; (7) radiotherapy or chemotherapy patients.

Individuals in the control group were selected from the outpatient clinics of the Oral Medicine Department. The selected controls had come for complaints that were not related to inflammatory diseases, and they had no evidence of hairy tongue lesions. The control exclusion criteria were the same as those for the main study group.

### 2.4. Outcome Measures

The patients were asked to wash their mouths with sterile water before performing the intraoral examination of the tongue. Moreover, they were asked to open their mouth and protrude the tongue as much as possible and were examined with sterile gloves. Tongue examination was done according to the guidelines of the WHO without performing any cytology or biopsy [26].

### 2.5. The First Outcome Variable: Hairy Tongue Index

The hairy tongue index was assessed by a visual method based on observations of the dorsum tongue appearance, as described by Shimizu et al. A score between 0 and 3 was given, with Score 0 indicating the absence of any tongue coating; Score 1: thin tongue coating and visible tongue papillae; Score 2: very thick tongue coating with visible tongue papillae; and Score 3: a hair-like appearance of the tongue with a dark color. If the examiner scored two or higher, participants were regarded as having hairy tongues [27].

### 2.6. The Second Outcome Variable: H. pylori IgG Antibodies:

Two mL of venous blood was collected by 5 mL disposable sterile syringes and poured into simple test tubes. The tubes were labeled with the subject’s name, age, and gender and sent to the laboratory within 1 h, where the blood was coagulated and the clot separated from the serum by centrifugation at a speed of 7000 rpm for 25 min. The resultant serum was transferred into 5 mL transparent plastic tubes and stored at −20 °C. This serological test is based on the serum levels of anti-*H pylori* IgG antibodies to detect Helicobacter pylori presence. Serological testing for the *H. pylori* IgG-antibodies was performed using a commercially available enzyme-linked immunosorbent assay by Immulite 2000 XPi (Immunoassay System, Siemens, Munich, Germany). According to the manufacturer’s guidelines, results were considered positive if the antibody titers were more than 1.1 U/mL. This assay has been assessed in a population similar to the presented trial and has a proven sensitivity of 98–100% and a specificity of 79–85% [28].

### 2.7. Statistical Analysis

All the data were analyzed using the Statistical Package for Social Science (SPSS) program (version 22.00; IBM Corp., Armonk, NY, USA). The mean age was compared using independent t-tests, while gender and other characteristics were compared using the Chi-square test. A *p*-value of less than 0.05 was considered statistically significant.

## 3. Results

The Chi-square test was used to associate the occurrence of the hairy tongue with age, gender, and *H. pylori* infections. Of a total of 40 patients, whose ages ranged from 20–79 years, 23 were males, whose mean age was 41.30 ± 13.55 years, and 17 were females, whose mean age was 41.59 ± 9.93 years). In the Cases group (n = 20), 15 (75%) patients were males and 5 (25%) were females; the mean age was 44.53 ± 13.255 and 46.8 ± 10.109, respectively. In the control group (n = 20), 8 (40%) patients were males and 12 (60%) were females; the mean age was 35.25 ± 12.703 and 39.42 ± 9.414 years, respectively (Table 1).

The prevalence of hairy tongue was higher among males, 15 (75.0%), compared to females, 5 (25.0%). This greater prevalence was found to be statistically significant (*p* = 0.026). In other words, with a 95% confidence level, there was a statistically significant association between gender and the presence of the hairy tongue lesion as it was found that the hairy tongue infection in males was more common than in females in a ratio of 3:1 (Table 2).

The hairy tongue lesions were found to be least in the 20–39 years of age group with 3 cases (15%), and it was most prevalent in the 40–59 age group with 10 cases (50%), followed by 60–79 years of age with 7 cases (35%), as compared to the control group, where the proportions were 60.0%, 35.0%, and 5.0% respectively. There was no statistically significant correlation between age groups and hairy tongues (Table 3).

*H. pylori* infection was detected positive in 14 patients (70%) and negative in six (30%) out of hairy tongue patients, compared to the control group, where the rates were 15% and 85%, respectively. In addition, it was revealed that 82.35% of the patients with hairy tongues had *H. pylori* infection, whereas this value was 17.65% in the participants with non-hairy tongues according to the positive *H. pylori* infection. The findings showed a statistically significant correlation between infection with *H-Pylori* and hairy tongue (*p* = 0.001; Table 4). *H-Pylori* was common in people with hairy tongues at 82.35% and the serum levels of anti-*H pylori*. IgG antibodies were increased in the presence of a hairy tongue by more than 1.1 U/mL.

## 4. Discussion

Tongue manifestation is an important pathophysiological indicator that Chinese practitioners have used to diagnose diseases for thousands of years [29]. The gender distribution of the current study showed that the HT lesions were additionally rife among males (75%) vs. females (25%) when put next with the management cluster (40%, 60%). It agrees that the majority of studies showed that males have a higher proportion of hairy tongue lesions as compared to female patients [1,30]. According to some reports, the hairy tongue was about thrice as common in men as in women [31,32]. This may be attributed to the greater prominence of smoking and poor oral hygiene in males [33]. On the other hand, this distinction is offset in Finland, in which smoking habits were declining amongst men, and younger ladies are not much more likely to be laid low with BHT, which is consistent with Vuorenkoski in 2008 [11], so the reasons for the diverse consequences stand out from the type of population and its etiology. 

Age may be a predisposing factor, with older males being a lot of possible to develop a black tongue lesion because the results were found in our study showed that the bulk of the age case cluster was in the 40–59 age group, with a share of 50%, followed by the 60–79 age group with 35%, and finally, the 20–39 age group with 15%, with an examination of (35%, 5%, 60%, respectively) in the control group. This agrees with most studies showing a prevalence of nearly 40% in patients over the age of sixty years [34], alternative studies considered black tongues a common occurrence in older patients [8,35], which is consistent with some studies. A hairy tongue is believed to be an illness that is most common in adulthood thanks to a reaction of acid within the mouth caused by gastritis, which is a far more likely cause than food or saliva in the aged. The acid reaction favors the expansion of yeasts and filament-like, acid-loving bacilli, and thus, the adhesion of the animal tissue scales to every other. This seems to be the essential underlying issue [30]. In contrast, some cases were noted in patients as young as 3 months [36] and 1-month-old [37]; however, these were uncommon reported cases.

Several studies discuss the link between gastrointestinal disorders and tongue features, including tongue-coating thickness, tongue-coating microbiom, metabolic markers, and tongue temperature [5]. However, no articles discussed the hairy tongue lesion and an independent disease that may be related to gastrointestinal diseases, particularly *H. pylori* infection. The results of this trial are expected to provide valuable evidence for the presence of hairy lesions on the tongue to assess the status of patients with *H.pylori* infection to help doctors identify potential problems and implement proper management of this condition. A change in glucose metabolism is one of the mechanisms of hairy tongue formation in sufferers with persistent gastritis, which shows that the occurrence of persistent gastritis is associated with strength metabolism adjustments and the microecological index of hairy tongue coating [38].

Our study investigated the correlation between *H-pylori* infection and the hairy tongue and suggested that the tongue could be a feasible predictor of *H-pylori* infection, especially in patients with gastric infection symptoms. According to some studies, it is believed that HT is due to the reaction of acid in the mouth produced by gastritis, an excess of fermentable food, or a lack of saliva in the aged. The acid reaction favors the growth of yeasts and filiform acidophilous bacilli, and the adhesion of the epithelial cells appears to be the important underlying factor [39]. Moreover, other studies suggested that *H-pylori* infection and gastritis in the corpus suppress acid secretion and increase gastric juice PH, resulting in hypergastrinemia, and that eradication of *H-pylori* normalizes acid secretion and serum gastrin levels [40].

However, several studies suggested that the relationship between *H-pylori* in the oral cavity and the stomach has garnered considerable interest from clinicians and scientists. However, they found *H-pylori* in different niches in the oral cavity, such as periodontal areas, mucosa, and saliva [41]. This agreed with Zou and Li’s study, which indicated that oral *H-pylori* and stomach Hp had a certain correlation. Also, Assumpção et al. showed that the *H-pylori* genotype in the oral cavity was found to be consistent with that in the gastric mucosa in 89% of patients, and the results by Wang et al. showed that 95% of at least one genotype in the dental plaque was consistent with that in the gastric mucosa and 27% of all genotypes in the dental plaque were consistent with those in the gastric mucosa [42]. Other studies considered the oral cavity as another primary storage of *H-pylori*, in addition to the stomach, and a certain homology exists between *H-pylori* in the oral cavity and Hp in the stomach. On the other hand, some articles improved oral *H-pylori* detection in patients with *H-pylori*-associated gastrointestinal diseases and proved the necessity and feasibility of the systematic eradication of stomach *H-pylori* infections and oral Hp infections. Furthermore, they suggested that the key to the successful eradication of gastric *H-pylori* is the ability to remove oral H-pylori completely [42].

Our findings are comparable to the results of studies among outpatients in Iran and China that noticed the changes in the color of the middle of the tongue with thick coating are indicators of a risk of *H-pylori* infection in patients with GI symptoms [21], and also suggested that the frequency of *H-pylori* infection was significantly increased in patients with red and purple tongues compared with those with pale red tongues [38].

Accordingly, we can consider the hairy tongue a noninvasive screening tool for the early diagnosis of *H-pylori*. As a result, the hairy tongue could be considered an indicator of *H. pylori* infection.

The current study has some limitations: Firstly, it is a single-center study based on a small number of patients, which limits the possibility of generalizing its findings. Secondly, the inclusion of other screening tests in the diagnosis of the infection of *H-pylori* was not performed. Thirdly, the analysis of the possible interactions of some confounding factors was not covered in this research work, such as tobacco smoking, the prolonged use of antibiotics, Candidiasis, xerostomia, or other diseases accused of causing the hairy tongue lesion. Due to the cross-sectional nature of this study, no casual association could be established. Finally, no multivariate analysis was applied, and this could be employed in future research work.

## 5. Conclusions

The hairy tongue might be considered an indicator of *H-pylori* infection. Therefore, dentists and internists need to pay more attention to the consistent hairy tongue to find *H-pylori* infection in the primary stages. Early diagnosis and treatment of this infection not only eliminates the effects of the hairy tongue but can also potentially prevent other gastric disorders and malignancies; focusing on the therapy of oral *H-pylori* can not only increase the eradication rate and reduce the recurrence rate of gastric *H-pylori* but can also play a protective role in oral care. Future studies need to investigate the presence of hairy tongues after the treatment of *H-pylori* infection and the effectiveness of combined treatment between the stomach and oral cavity.

## Figures and Tables

**Table 1:** Descriptive statistics of the demographic variables for the study sample (n = 40).

Variable		Hairy Tongue Group		Control Group		Full Sample	
		Frequency	%	Frequency	%	Frequency	%
Gender	Male	15	75.0	8	40.0	23	57.5
	Female	5	25.0	12	60.0	17	42.5
	Total	20	100.0	20	100.0	40	100.0
Age group	20 to 39	7	35.0	12	60.0	19	47.5
	40 to 59	10	50.0	7	35.0	17	42.5
	60 to 79	3	15.0	1	5.0	4	10.0
	Total	20	100.0	20	100.0	40	100.0

**Table 2:** Distribution of the hairy tongue lesions according to the gender in the hairy tongue group and the control group.

Group	Gender		*p*-Value ^†^
	Male Frequency (%)	Female Frequency (%)	
Hairy tongue	15 (65.2)	5 (29.4)	0.026 *
Control	7 (34.8)	13 (70.6)	

^†^ Chi-square test was used, * significant at *p* < 0.05; the chi-square statistic with Yates’ correction was 4.9495.

**Table 3:** Distribution of the hairy tongue lesions according to the age groups in the hairy tongue group and the control group with the result of significance testing.

Group	Age Groups			*p*-Value ^†^
	20–39 Years Frequency (%)	40–59 Years Frequency (%)	60–79 Years Frequency (%)	
Hairy tongue	7 (36.8)	10 (58.8)	3 (75.0)	0.241 (NS)
Control	12 (63.2)	7 (41.2)	1 (25.0)	

^†^ Chi-square test was used; NS: non-significant difference; the chi-square statistic was 2.8452.

**Table 4:** Evaluation of the association between the presence of the hairy tongue lesion and the infection of the H-Pylori.

Variable	Group	H-Pylori Infection		*p*-Value ^†^
		No Frequency (%)	Yes Frequency (%)	
Group	Hairy tongue	6 (26.09%)	14 (82.35%)	0.001 *
	Control	17 (73.91%)	3 (17.65%)	

^†^ Chi-square test was used; * significant at *p* < 0.05. The chi-square statistic with Yates correction was 10.2302.

## Data Availability

The data presented in this study are available on request from the corresponding author.

## References

[1] Al-Wesabi M.A., Al-Hajri M., Shamala A., Al-Sanaani S. (2017). Tongue lesions and anomalies in a sample of Yemeni dental patients: A cross-sectional study. J. Oral Res..

[2] Anand P.S., Kamath K.P., Anil S. (2014). Role of dental plaque, saliva and periodontal disease in Helicobacter pylori infection. World J. Gastroenterol. WJG.

[3] Anitha N., Jayachandran D. (2021). Tongue Lesions-A Review. Nveo-Nat. Volatiles Essent. OILS J..

[4] Avellaneda C.B., Inzunza A.F.B. (2021). Lengua negra pilosa asociada a carcinoma escamocelular de esófago. Rev. Colomb. de Gastroenterol..

[5] Bordoni B., Morabito B., Mitrano R., Simonelli M., Toccafondi A. (2018). The Anatomical Relationships of the Tongue with the Body System. Cureus.

[6] Boyanova L., Panov V., Yordanov D., Gergova G., Mitov I. (2013). Characterization of oral Helicobacter pylori strain by 4 methods. Diagn. Microbiol. Infect. Dis..

[7] Burge E., Kogilwaimath S. (2021). Hairy tongue. Can. Med. Assoc. J..

[8] Casu C., Nosotti M.G., Sinesi A. (2019). Hairy tongue, geographic tongue, scrotal tongue and systemic connections: Clinical images and an overview. Dent. Case Rep..

[9] Darwazeh A., Almelaih A. (2011). Tongue lesions in a Jordanian population. Prevalence, symptoms, subject s knowledge and treatment provided. Med. Oral Patol. Oral Cir. Bucal..

[10] Diaconu S., Predescu A., Moldoveanu A., Pop C., Fierbințeanu-Braticevici C. (2017). Helicobacter pylori infection: Old and new. J. Med. Life.

[11] Dwivedi V., Torwane N.A., Tyagi S., Maran S. (2019). Effectiveness of Various Tongue Cleaning Aids in the Reduction of Tongue Coating and Bacterial Load: A Comparative Clinical Study. J. Contemp. Dent. Pract..

[12] Feng H. (2018). Research progress in thecorrelation between oral and stomach Hpinfections. Infect. Int..

[13] Gurvits G.E., Tan A. (2014). Black hairy tongue syndrome. World J. Gastroenterol..

[14] Hajifattahi F., Hesari M., Zojaji H., Sarlati F. (2015). Relationship of Halitosis with Gastric Helicobacter Pylori Infection. J. Dent. Tehran Univ. Med. Sci..

[15] Hou B.-N., Zeng Y.-D., Liang H., Liu R., Zhou X.-Q., Zhang L., Peng Q.-H. (2018). Correlation Between Helicobacter pylori Infection and Tongue Manifestations: A Meta-analysis. Digit. Chin. Med..

[16] Kainuma M., Furusyo N., Urita Y., Nagata M., Ihara T., Oji T., Nakaguchi T., Namiki T., Hayashi J. (2015). The association between objective tongue color and endoscopic findings: Results from the Kyushu and Okinawa population study (KOPS). BMC Complement. Altern. Med..

[17] Lamster I.B. (2021). The 2021 WHO Resolution on Oral Health. Int. Dent. J..

[18] Locatelli A., Catapani W.R., Junior C.R.G., Silva C.B.P., Waisberg J. (2004). Detection of anti-Helicobacter pyloriantibodies in serum and duodenal fluid in peptic gastroduodenal disease. World J. Gastroenterol..

[19] Mohammadi K., Sharifi P., Ataee P., Pakanzad S.F., Reshadmanesh N., Gharibi F., Moradsalimi E., Ghafouri H. (2019). Evaluation of Helicobacter pylori Infection and Its Relation with Coated Tongue in Patients Referring to UBT. J. Ilam Univ. Med. Sci..

[20] Murry T., Carrau R.L., Chan K. (2020). Clinical Management of Swallowing Disorders.

[21] Owczarek-Drabińska J.E., Radwan-Oczko M. (2020). A Case of Lingua Villosa Nigra (Black Hairy Tongue) in a 3-Month-Old Infant. Am. J. Case Rep..

[22] Pandya H.B., Patel J.S., Agravat H.H. (2014). Noninvasive diagnosis of Helicobacter pylori: Evaluation of two enzyme immunoassays, testing serum IgG and IgA response in the Anand district of central Gujarat, India. J. Clin. Diagn. Res. JCDR.

[23] Popik E., Barroso F., Pombeiro J., Carvalho C., Almeida A. (2018). Hairy tongue in a 1-month-old infant. Arch. Dis. Child..

[24] Arora R., Prabha N., Singh N., Nagarkar N. (2020). Glycopyrrolate-induced black hairy tongue. Indian Dermatol. Online J..

[25] Rajarammohan K., Narayanan M., Ravikumar P.T., Fenn S.M., Gokulraj S. (2020). Hairy Tongue—A Series of 4 Cases. J. Evol. Med. Dent. Sci..

[26] Ramanathan J., Leclercq M.H., Mendis B.R., Barmes D.E. (1995). Gathering data on oral mucosal diseases: A new approach. World Health Forum.

[27] Rosebush M.S., Briody A.N., Cordell K.G. (2019). Black and Brown: Non-neoplastic Pigmentation of the Oral Mucosa. Head Neck Pathol..

[28] Schlager E., Claire C.S., Ashack K., Khachemoune A. (2017). Black Hairy Tongue: Predisposing Factors, Diagnosis, and Treatment. Am. J. Clin. Dermatol..

[29] Shayeb M.A., Fathy E., Nadeem G., El-Sahn N.A., Elsahn H., Khader I.E., Habbal A.W.A. (2020). Prevalence of most common tongue lesions among a group of UAE population: Retrospective study. Onkol. Radioter..

[30] Shimizu T., Ueda T., Sakurai K. (2007). New method for evaluation of tongue-coating status. J. Oral Rehabil..

[31] Sun Z.-M., Zhao J., Qian P., Wang Y.-Q., Zhang W.-F., Guo C.-R., Pang X.-Y., Wang S.-C., Li F.-F., Li Q. (2013). Metabolic markers and microecological characteristics of tongue coating in patients with chronic gastritis. BMC Complement. Altern. Med..

[32] Suzuki N., Beppu R., Yoneda M., Takeshita T., Asakawa M., Yamashita Y., Hanioka T., Hirofuji T., Shinohara T. (2020). Effects of eradication of Helicobacter pylori on oral malodor and the oral environment: A single-center observational study. BMC Res. Notes.

[33] Furuta S.B.T. (1998). Effect of *Helicobacter pylori* Infection on Gastric Juice pH. Scand. J. Gastroenterol..

[34] Talebi Bezmin Abadi A. (2018). Diagnosis of Helicobacter pylori Using Invasive and Noninvasive Approaches. J. Pathog..

[35] Vonkeman H.E., Deleest H., Van Delaar M., Vanbaarlen J., Steen K., Lems W., Bijlsma J., Kuipers E., Houben H., Janssen M. (2012). Assessment of Helicobacter pylorieradication in patients on NSAID treatment. BMC Gastroenterol..

[36] Wan X., Tang D., Zhang X., Li H., Cui Z., Hu S., Huang M. (2014). Exploratory study of oral mucosal colonization of human gastric Helicobacter pylori in mice. Int. J. Clin. Exp. Med..

[37] Wang H.-H., Pan C.-H., Wu P.-P., Luo S.-F., Lin H.-J., Wu C.-H. (2012). Alteration of the Tongue Manifestation Reflects Clinical Outcomes of Peptic Ulcer Disease. J. Altern. Complement. Med..

[38] Wang X.M., Yee K.C., Hazeki-Taylor N., Li J., Fu H.Y., Huang M.L., Zhang G.Y. (2014). Oral Helicobacter pylori, its relationship to successful eradication of gastric H. pylori and saliva culture confirmation. J. Physiol. Pharmacol. Off. J. Pol. Physiol. Soc..

[39] Wu Z., Zou K., Xiang C., Jin Z., Ding H., Xu S., Wu G., Wang Y., Wu X., Chen C. (2021). *Helicobacter pylori* infection is associated with the co-occurrence of bacteria in the oral cavity and the gastric mucosa. Helicobacter.

[40] Yadav R., Sagar M. (2022). Comparison of Different Histological Staining Methods for Detection of Helicobacter pylori Infection in Gastric Biopsy. Cureus.

[41] Zaric S., Bojic B., Popović B., Milasin J. (2015). Eradication of Gastric Helicobacter pylori ameliorates Halitosis and Tongue Coating. J. Contemp. Dent. Pract..

[42] Zheng Y., Liu M., Shu H., Chen Z., Liu G., Zhang Y. (2014). Relationship between oral problems and Helicobacter pylori infection. Arch. Oral Biol..

